# Functional outcomes among stroke patients in Alagoas, Brazil: observational study

**DOI:** 10.1590/1516-3180.2020.0304.r2.10122020

**Published:** 2021-03-12

**Authors:** Jussara Almeida de Oliveira Baggio, Dandhara Henrique de Farias, Lizanilda Leite de Gusmão Albuquerque, Bianca Cardoso de Melo, Valquíria da Silva, Daniela Bassi-Dibai, Letícia Januzi de Almeida Rocha

**Affiliations:** I PhD. Physiotherapist and Assistant Professor, Universidade Federal de Alagoas (UFAL), Arapiraca (AL), Brazil.; II Undergraduate Student, Centro Universitário Tiradentes, Maceió (AL), Brazil.; III Undergraduate Student, Centro Universitário Tiradentes, Maceió (AL), Brazil.; IV Undergraduate Student, Centro Universitário Tiradentes, Maceió (AL), Brazil.; V Undergraduate Student, Centro Universitário Tiradentes, Maceió (AL), Brazil.; VI PhD. Physiotherapist and Professor at Master's Program on Management of Health Services, Universidade Ceuma, São Luís (MA), Brazil.; VII MD, MSc. Physician and Professor, Faculdade de Medicina da Universidade Federal de Alagoas (UFAL), Maceió (AL), Brazil.

**Keywords:** Stroke rehabilitation, Stroke, Disabled persons, Rehabilitation, Cerebrovascular disease, Functional outcome, Rehabilitation process

## Abstract

**BACKGROUND:**

Stroke is the principal cause of disability around the world and the ensuing functional dependence (FD) can be correlated with different factors.

**OBJECTIVE:**

To determine how demographic factors and clinical characteristics after stroke distinguish patients who achieve functional independence from those who do not.

**DESIGN AND SETTING:**

Observational study at specialized neurovascular clinic in Alagoas, Brazil.

**METHODS:**

FD was classified according to the modified Rankin scale (mRs): 0 to 2 points were classified as independent (FD-), and 3 to 5 points were classified as dependent (FD+). Logistic regression analysis included age, sedentary lifestyle, the Center for Epidemiological Studies – Depression Scale (CES-D) and the National Institutes of Health Stroke Scale (NIHSS). The Mann-Whitney test and χ^2^ test were used to compare groups.

**RESULTS:**

We included 190 stroke patients with a mean age of 60.02 ± 14.22 years. We found that 34.8% of the patients were classified as FD+. Lower NIHSS and CES-D scores were more associated with achieving functional independence. Most of the patients had access to physical therapy, and the mean duration of rehabilitation therapy was 65.2 minutes per week. Females had higher prevalence of depressive symptoms (P = 0.005) and rehabilitation time was shorter for hemorrhagic stroke (P = 0.02).

**CONCLUSION:**

We found a FD rate four times greater than in another Brazilian study. Lower stroke severity and fewer depressive symptoms were associated with achieving functional independence. Less than half of the patients were referred to a rehabilitation service at hospital discharge and few had access to multidisciplinary treatment.

## INTRODUCTION

Stroke is an epidemic disease. Up to 2013, there had been more than 25 million stroke survivors and more than 6 million deaths worldwide.^1^ In Brazil, more than 2 million cases of stroke were reported in 2013, with approximately 25% of these individuals remaining disabled.^2^ Estimates from the Global Burden Disease Study showed prevalence of 170-339/100,000 for ischemic stroke and 0-39/100,000 for hemorrhagic stroke in Brazil.^3^

Despite these impactful numbers, reductions in the mortality rates due to stroke have been achieved in developing countries over the past two decades.^1^ A study conducted in the city of Joinville, in southern Brazil, showed a significant reduction in incidence of 37%. These results can be explained by improvements in socioeconomic conditions and educational levels in this city.^4^

However, Brazil is a country of continental dimensions, and the reality is not the same throughout the national territory. Until now, little information about stroke in northeastern Brazil has been available. A study conducted in Fortaleza, in 2011, showed a prevalence of risk factors similar to what has been reported in the literature, including both high functional dependence (FD) after stroke and high in-hospital mortality.^5^ A comparison among all the state capitals of northeastern Brazil showed that Maceio, state of Alagoas, had the highest mortality rate due to stroke and the lowest human development index (HDI).^6^ Because of this, it is important to know more about the characteristics of stroke in this region.

In Brazil, information about the level of FD after stroke and the characteristics of these patients remains scarce. In the United States, stroke is the main cause of disability,^7^ while in Brazil, 15%-20% of stroke patients have neurological deficits in the first year and 10% of ischemic stroke survivors continue to have FD three years after stroke.^8^ Thus, we hypothesize that stroke patients in Alagoas will have high levels of FD.

## OBJECTIVE

The main objective of this study was to determine how demographic factors and clinical characteristics after stroke distinguish patients who achieve functional independence from those who do not, in a sample of patients attended at a specialized neurovascular clinic in Alagoas, Brazil. We also compared functionality in ischemic and hemorrhagic stroke patients and characterized the rehabilitation process of these patients.

## METHODS

### Design and ethical aspects

This was an observational study. The study procedures were approved by the research ethics committee of the local institution, in accordance with opinion report number 60717416.1.0000.5013 (October 27, 2016). Written informed consent was obtained from all of the participants.

### Sample

We evaluated patients with ischemic and hemorrhagic stroke confirmed by neuroimaging who were consecutively attended at a specialized neurovascular clinic in Alagoas, Brazil. Patients were excluded if they were younger than 18 years old, had another neurological disease associated with stroke or had a severe concomitant systemic illness, such as cancer, severe kidney disease or severe hepatic disease.

### Initial evaluation

The protocol assessment was composed of demographic and clinical data. To evaluate stroke severity, we used the National Institutes of Health Stroke Scale (NIHSS), which is composed of 11 items and has a maximum score of 42. Higher scores on this scale mean worse stroke severity.

To measure functional status, we used the Modified Rankin Scale (mRs) and the Functional Independence Measure (FIM). The mRs is often used to measure overall disability and primary outcomes in randomized clinical trials,^9^ and its scale ranges from 0 (no symptom) to 6 (death). The FIM was developed to evaluate patients with different diagnostic and functional restrictions, and the main objective is to measure the burden of care demanded by a person in order to execute a series of motor and cognitive tasks.^10^ The scale contains 18 items and is rated on a 7-point scale, ranging from 1 (total assistance needed) to 7 (complete independence).

The patients were also evaluated using the Center for Epidemiological Studies – Depression Scale (CES-D),^11^ which is composed of 20 items. On this scale, higher scores suggest depressive symptoms. Furthermore, a structured questionnaire about implementation, frequency and duration of rehabilitation therapies was applied.

We classified functionality using the mRs. On this scale, patients with 0 to 2 points were classified as independent (FD-), and those with 3 to 5 points were classified as dependent (FD+).

### Statistical analysis

All data were analyzed using the SPSS software (version 20.0; Chicago, IL, United States) at a significance level of 5%.

The distribution of the data was initially verified by means of histogram analysis. We used descriptive statistics to show the demographic and clinical characteristics of stroke patients and the proportion of FD based on the mRs and FIM scores.

Logistic regression analysis was performed using a dichotomous dependent variable (FD+ = 0 and FD- = 1), with the following admission variables to predict group associations: age, sedentary lifestyle, CES-D and NIHSS. The variables of age, CES-D and NIHSS were classified as continuous and the variable of sedentary lifestyle was classified as categorical. These were entered into the logistic regression to confirm the final variables in the model. The Hosmer and Lemeshow test confirmed that all the regression models adequately fitted the data (P > 0.05).

For descriptive purposes, the Mann Whitney test and χ^2^ test were used to compare the groups (FD+ and FD-). The variables with statistical significance were chosen for the logistic regression. We also compared groups of ischemic versus hemorrhagic stroke and of female versus male patients, using the Mann-Whitney test and χ^2^ test.

## RESULTS

A total of 190 stroke patients with a mean age of 60.02 ± 14.22 years were evaluated. Among these patients, 61.6% were male and the mean schooling level was 5.8 ± 4.4 years. The mean time that had elapsed from the stroke to inclusion in the study was 27.2 ± 33.1 months. The demographic and clinical characteristics of these stroke patients are described in **Table 1**.

**Table 1:** Demographic and clinical characteristics of all stroke patients and according to whether they were classified as presenting functional dependence or functional independence

Variables		All stroke patients (n = 190)	Patients with functional dependence* (n = 68)	Patients with functional independence* (n = 122)	P-value
**Age (years)**		60.02 ± 14.22	62.69 ± 1.71	58.56 ± 1.28	0.017#
**Male (%)**		117 (61.6%)	36 (52.9%)	81 (66.3%)	0.08
**Schooling level**		5.86 ± 4.44	5.22 ± 0.51	6.20 ± 0.41	0.14
**Marital status (%)**					
	Married	116 (61.05%)	40 (58.8%)	76 (62.2%)	
	Single	37 (19.4%)	14 (20.5%)	23 (18.8%)	0.4
	Widowed	9 (4.7%)	1 (1.4%)	8 (6.5%)	
	Divorced	28 (14.7%)	13 (19.1%)	15 (12.2%)	
**Income**					
**(in minimum monthly wages)**					
	Up to 1	64 (33.7%)	19 (27.9%)	45 (36.8%)	0.3
	2 to 3	84 (44.2%)	33 (48.5%)	51 (41.8%)	
	> 3	17 (8.9%)	4 (5.8%)	13 (10.6%)	
	Not declared	25 (13.2%)	12 (17.6%)	13 (10.6%)	
**Stroke type**					0.06
	Ischemic	168 (88.4%)	66 (97%)	102 (83.6%)	
	Hemorrhagic	16 (8.4%)	2 (2.9%)	14 (11.4%)	
	Both	6 (3.1%)	0 (0%)	6 (4.9%)	
**Risk factors**					
	Sedentary lifestyle (yes)	118 (62.1%)	48 (70.5%)	70 (57.3%)	0.004##
	Smoking (yes)	40 (21.1%)	9 (13.2%)	31 (25.4%)	0.06
	Alcohol consumption (yes)	59 (31.1%)	19 (27.9%)	40 (32.7%)	0.51
	High blood pressure (yes)	150 (78.9%)	56 (82.3%)	95 (77.8%)	0.57
	Diabetes mellitus (yes)	72 (37.9%)	25 (36.7%)	47 (38.5%)	0.87
	Cardiopathy associated (yes)	48 (25.3%)	21 (30.8%)	27 (22.1%)	0.22
**NIHSS**		2 (IQR: 1-5)	5 (IQR: 1-7)	1 (IQR: 0-3)	0.0001#
**CES-D**		15.7 ± 11.6	18.88 ± 1.8	14.56 ± 1.05	0.008#
**FIM total**		99.1 ± 26.3	80.13 ± 3.22	113.8 ± 1.16	0.0001#

FIM = Functional Independence Measure; NIHSS = National Institutes of Health Stroke Scale; CES-D = Center for Epidemiological Studies - Depression Scale.

* Functional dependence = patients with 3 to 5 points on modified Rankin scale; Functional independence = patients with 0 to 2 points on modified Rankin scale; IQR = interquartile range;

# Mann-Whitney test: significance level < 0.05;

## χ^2^ test: significance level < 0.05.

The mean CES-D score was 15.7 ± 11.6. Using the cutoff of 16 points, we found that 66 patients (40%) presented depressive symptoms. The mean FIM score was 99.1 ± 26.3. The median NIHSS score was 2 (interquartile range, IQR: 1-5), while the median score of the mRs was 2 (IQR: 1-3). We found that 34.8% of the patients were classified as FD+, according to the mRs. **Table 1** also compares the two groups (FD+ and FD-) and **Table 2** describes the results on the FIM scale, detailed according to item.

**Table 2:** Functional classification of stroke patients based on Functional Independence Measure (FIM) score*

Self-care		Functional dependence	Functional independence
**Eating**		76 (40%)	114 (60%)
**Grooming**		51 (26.8%)	139 (73.2%)
**Bathing**		58 (30.5%)	132 (69.5%)
**Dressing - upper body**		57 (30%)	133 (70%)
**Dressing - lower body**		60 (31.6%)	130 (68.4%)
**Toileting**		51 (26.8%)	139 (73.2%)
**Sphincter control**			
	Bladder management	61 (32.1%)	129 (67.9%)
	Bowel management	25 (13.2%)	165 (86.9%)
**Transfers**			
	Bed/chair/wheelchair	57 (30%)	133 (70%)
	Toilet	53 (28%)	137 (72%)
	Tub/shower	53 (28%)	137 (72%)
**Locomotion**			
	Walking/wheelchair	73 (38.4%)	117 (61.6%)
	Stairs	102 (53.7%)	88 (46.3%)
**Communication**			
	Comprehension	45 (23.7%)	145 (76.3%)
	Expression	65 (34.2%)	125 (65.8%)
**Social cognition**			
	Social interaction	61 (32.1%)	129 (67.9%)
	Problem solving	96 (50.5%)	94 (49.5%)
	Memory	87 (45.8%)	103 (51.2%)

* FIM score: 1 to 5 was classified as dependent, and 6 and 7 as independent.

We also investigated the rehabilitation process. Only 30% of the patients reported having undergone some type of rehabilitation therapy during the period of their hospital stay, while 41% were referred to a rehabilitation service at hospital discharge. At the time of the present evaluation, 41% had undergone rehabilitation therapy, including 40% receiving physical therapy, 11.6% speech therapy, 10% occupational therapy, 9.5% psychology and 7.4% nutritional monitoring. Of these patients, 44% underwent rehabilitation for more than 12 months, 25.3% for 3 to 12 months and 30.7% for less than 3 months. As many as 62.7% of the patients underwent rehabilitation twice a week, and the mean duration of their rehabilitation sessions was 65.2 minutes per week, with a range from 15 to 480 minutes.

**Table 3** describes the comparison between patients with ischemic and hemorrhagic stroke. We also compared the same variables between males and females, and only found a significant difference in CES-D score (P = 0.005). Additionally, the results from the logistic regression analysis are shown in **Table 4**. The model was significant, categorizing 77.8% correctly. Lower NIHSS and CES-D scores were more associated with achieving functional independence.

**Table 3:** Comparison between patients with ischemic and hemorrhagic stroke

	Ischemic stroke (n = 168)	Hemorrhagic stroke (n = 16)	P-value
Age (years)	58.48 ± 2.08	52.75 ± 5.58	0.34
Schooling level	6.05 ± 0.58	9.25 ± 1.75	0.13
NIHSS	3 (1-5)	2 (1-4)	0.44
mRs	2 (1-3)	2 (1-2)	0.47
FIM	100.21 ± 3.07	115.5 ± 1.7	0.25
CES-D	15.4 ± 1.47	11 ± 4.34	0.32
Rehabilitation therapy (yes)	70 (41.6%)	4 (25%)	0.15
Duration of rehabilitation (min/per week)	69.57 ± 9.56	28.75 ± 7.73	0.02*

NIHSS = National Institutes of Health Stroke Scale; mRs = modified Rankin scale; FIM = Functional Independence Measure; CES-D = Center for Epidemiological Studies - Depression Scale.

* Mann-Whitney test: significance level < 0.05.

**Table 4:** Results from logistic regression analysis (dependent variable is mRs score of 0-2; FD-)

Independent variable	B	SE	Wald	*df*	P	Odds ratio	95% CI
Age (years)	-0.12	0.016	0.53	1	0.46	0.98	0.95-1.02
Sedentary lifestyle (yes)	0.64	0.55	1.50	1	0.22	1.96	0.66-5.76
CES-D score	-0.38	0.019	4.17	1	0.04	0.96	0.92-0.99
NIHSS score	-0.52	0.10	25.68	1	0.0001	0.59	0.48-0.72
Constant	3.68	1.17	9.78	1	0.002	39.85	
Model X^2^	54.25, *df* = 4, P = 0.0001						
Pseudo R^2^	0.44						
N	144						
Hosmer and Lemeshow test (χ^2^)	10.05, *df* = 8; P = 0.26						

mRs = modified Rankin scale; NIHSS = National Institutes of Health Stroke Scale; CES-D = Center for Epidemiological Studies - Depression Scale; SE = standard error, Wald = Wald statistics; Pseudo R^2^ = Nagelkerke R square; *df* = degrees of freedom; CI = confidence interval.

Follow-ups on the patients were made using the mRs six months and one year after the patients had been included in the study. We were able to evaluate 82 patients after six months and 72 patients after one year. According to the mRs, after six months, 31.7% of the patients were classified as FD+, while after one year, 19.4% of patients were classified as FD+.

## DISCUSSION

To the best of our knowledge, this is the first study to evaluate functional outcomes among stroke patients living in the community in the state of Alagoas, Brazil, and the relationship between demographic factors and clinical characteristics after stroke that leads to achievement of functional independence. A detailed analysis according to state and region is necessary in a country like Brazil with huge socioeconomic differences. Moreover, a recent study showed that Maceió has the highest stroke mortality rate and lowest human development index (HDI) of all the state capitals in northeastern Brazil,^6^ which can directly influence the functional outcomes of stroke survivors.^12^

The mean time that had elapsed from the stroke to inclusion in the study was more than two years. This shows the difficulty that stroke patients have in accessing specialized neurovascular clinics after hospital discharge. This is largely because, in the whole state of Alagoas, there is only one center that specializes in monitoring stroke patients.

The CES-D is a screening tool for depression symptoms and its use in evaluating the emotional and cognitive status of stroke patients has been recommended by the National Institute of Neurological Disorders and Stroke (NINDS). A prevalence of depressive symptoms of 40% was found in our sample using the cutoff of 16 points, which was similar to findings from previous studies, in which prevalences of around 30%-40% were reported.^13^^–^^15^ Post-stroke depression (PSD) is multifactorial,^16^ and FD is one of its predictors.^15^ Our sample was composed of chronic patients with higher levels of FD, which explains the high prevalence of PSD.

Two different scales for analyzing functionality were selected. The mRs is the primary outcome scale for almost all clinical trials^17^^,^^18^ and is a validated instrument for assessing new stroke treatments.^9^ Frequently, it is analyzed dichotomously (scores of 0-2 versus 3-6). However, some authors have argued in favor of using the entire range of the scale, which could improve its statistical power and enable a more detailed analysis of disability.^9^^,^^19^^,^^20^ One negative point of the mRs is that it only has limited use in the field of rehabilitation, since this scale does not cover important aspects of rehabilitation, such as pain, communication and cognition.^21^ Because of this, we also used the FIM to complement the mRs results.

We found high levels of FD (34.8%) in our sample. Another Brazilian study found FD of 33%, 30 days after the ictus; 12% after 1 year; 9% after 2 years; and 8% after 3 years.^8^ Data from different countries have shown FD levels ranging from 20% to 52%.^22^^–^^24^ Greater stroke severity at the initial presentation, cardioembolic and large strokes and absence of adequate treatment in the acute phase have previously been reported to be related to worse functional outcomes. In fact, few patients with ischemic stroke in Alagoas had access to thrombolysis or thrombectomy. In the public healthcare system of Alagoas, there is only one stroke center for acute-phase treatment, while in its private healthcare system, only a few hospitals have instituted a stroke protocol. Moreover, it is important to note that these hospitals are located in the state capital (city of Maceió), and patients from other cities in Alagoas need to come to the capital to have access to these treatments.

The lack of recommended treatments described in the literature, for the acute phase of stroke, partially explains the occurrences of FD. After hospital discharge, the rehabilitation process continues, but less than half of these patients in Alagoas were referred to a rehabilitation service (41%). In the United States, more than two-thirds of stroke survivors receive rehabilitation after hospital discharge.^25^ In Australia, a study investigated the hospitals that participated in the 2015 National Stroke Audit, and out of 3,462 patients, 39.2% receive post-acute rehabilitation, which was a proportion similar to that of our study. However, 71.3% of those patients were treated in a stroke unit, 6.1% received thrombolysis and 72.4% received rehabilitation during acute hospitalization, which may have influenced the functionality of these patients and may have explained why a smaller number of patients needed rehabilitation. In the city of João Pessoa, Paraiba, also in northeastern Brazil, 67.1% of the patients received rehabilitation after stroke.^26^ Furthermore, in our study, few patients had access to speech therapy, occupational therapy or psychological care, which are fundamental for rehabilitation, since high levels of FD, in our sample, were related to poor levels of social cognition and poor levels of coping with activities of daily living.

In our study, we found that lower scores for NIHSS and CES-D were associated with functional independence after stroke. Higher stroke severity and greater numbers of depressive symptoms had already been pointed out in previous studies as potential barriers against achieving functional independence.^27^^–^^29^ However, these results emphasize that there is a need for public policies in the state of Alagoas, for increasing access to treatment for the acute phase of stroke and rehabilitation.

Another important finding from our study was that the mean duration of rehabilitation sessions was 65.2 minutes per week, with a significant difference between ischemic and hemorrhagic stroke patients. Although hemorrhagic stroke has a worse prognosis, most patients in our sample were considered functionally independent (87.5%) and, at the time evaluated in our study, 25% of them underwent rehabilitation.

In comparing male and female patients, it was seen in our study that the females had higher prevalence of depressive symptoms (p = 0.005). In the literature, the relationship between sex and depression among stroke patients is not consistent. Some previous studies had already shown that female gender was an independent risk factor for PSD in the acute phase and subacute phase.^30^^,^^31^ In addition, in the chronic phase, male gender was associated with depression.^32^^,^^33^ However, in a meta-analysis, age at the time of the study and gender were not predictors of depression.^34^

The ideal amount of rehabilitation is still a matter for debate. The latest stroke rehabilitation guidelines recommend use of intensive and repetitive functional tasks for training gait and for diminishing upper-limb limitations (level 1A of evidence). However, the ideal dose and frequency have not been determined.^35^ In the acute phase of stroke, high amounts (minutes per day) of mobilization have been shown to reduce positive functional outcomes, while increasing the daily frequency of out-of-bed sessions improved functional outcomes three months after stroke.^36^ In the subacute and chronic phases of stroke, high intensities of rehabilitation were correlated with preventing recurrent stroke and mortality.^37^ Also, increasing the total duration of physical therapy was associated with significant changes to motor FIM.^38^ Other factors, such as socioeconomic factors, pre-existing comorbidities and the skill of the rehabilitation team, which were not investigated in the present study, may have had an influence on the functional outcomes of these patients and need to be better understood in samples of this nature.

## CONCLUSION

We found a FD rate that was four times greater than what had been observed in another Brazilian study,^8^ and this rate was associated with higher stroke severity and greater numbers of depressive symptoms. These results can be explained by the restricted access to treatment in the acute phase of stroke that is available in the state of Alagoas. Another important matter was the rehabilitation process, considering that less than half of the patients were referred to a rehabilitation service at hospital discharge and few had access to multidisciplinary treatment. Moreover, the time per week dedicated to the rehabilitation therapies was low. These results reflect the precarious organization of stroke care in Alagoas and raises questions about how this state's rehabilitation services are organized and whether treatments are being offered in accordance with international recommendations.
